# Self-Construal Priming Affects Holistic Face Processing and Race Categorization, but Not Face Recognition

**DOI:** 10.3389/fpsyg.2019.01973

**Published:** 2019-08-27

**Authors:** Xinge Liu, Xingfen Liang, Cong Feng, Guomei Zhou

**Affiliations:** ^1^Department of Psychology, Sun Yat-sen University, Guangzhou, China; ^2^Department of Philosophy, Sun Yat-sen University, Guangzhou, China; ^3^Guangdong Provincial Key Laboratory of Social Cognitive Neuroscience and Mental Health, Sun Yat-sen University, Guangzhou, China

**Keywords:** self-construal priming, composite face effect, holistic processing, race, face recognition

## Abstract

Self-construal priming can affect an individual’s cognitive processing. Participants who were primed with interdependent self-construal showed more holistic process bias than those who were primed with independent self-construal. The holistic processing of a face also differs across cultures. As such, the purpose of the present study was to explore whether the cultural differences in holistic face processing can be interpreted from the perspective of self-construal, as well as to investigate the relationship between self-construal and holistic face processing/face recognition/race categorization. In Experiment 1, participants were primed with control, interdependent, or independent self-construal, respectively, and then they completed a feature-space same-different task (Experiment 1A) or a composite face effect task (Experiment 1B). Results showed no priming effect in Experiment 1A, whereas independent self-construal priming resulted in less holistic processing in Experiment 1B. In Experiment 2, participants were primed with control, collective/interdependent, relational, or independent self-construal, respectively, and then they completed a Vanderbilt Holistic Face Processing Test and Cambridge Face Memory Test. Participants who were primed as independent showed greater congruency effect than the relational group. Self-construal priming had no effect on face recognition. In Experiment 3, we manipulated self-construal in the same way as that in Experiment 2 and monitored the eye movement of Chinese participants while they learned, recognized, and categorized their own-/other-race faces. Self-construal priming had no effect on face recognition. Compared with other groups, collective-/interdependent-self priming increased the fixation time of eyes and decreased the fixation time of nose in the race categorization task. These results indicated that the cultural differences in self-construal could not mirror the cultural differences in face processing in a simple way.

## Introduction

### Cultural Difference in Self-Construal

Markus and Kitayama (1991) first proposed two kinds of self-construal: independent self and interdependent self. The following descriptions of these two kinds of self-construal are summarized from Markus and Kitayama (1991) and Singelis (1994). Independent self emphasizes the individual’s uniqueness. People with such self-construal tend to separate themselves from the social background. When thinking about themselves, individuals with high independent self-construal will be more inclined to express their own abilities, traits, characteristics, or goals, rather than the feelings, thoughts, or actions of others. Interdependent self builds on relationships, identities, social status, and external expectations, emphasizing the connectedness and collectivity of individuals with others and their social relationships. Self-construal varies across cultures, being more independent in Western cultures and interdependent in Eastern cultures (Markus and Kitayama, 1991; Markus et al., 1996).

Some theories implicitly proposed further distinction between two levels of interdependent self: the relational self that derives from interpersonal relationships and interdependence with specific others, as well as the collective self that derive from membership in larger, more impersonal collectives, or social categories (see Brewer and Gardner, 1996; Brewer and Chen, 2007; for a review). In the tripartite model, the self comprises three levels of self-construal: individual, relational, and collective (Brewer and Gardner, 1996; Brewer and Chen, 2007; Tanti et al., 2008; Kashima et al., 2011; Sedikides et al., 2011; Lee et al., 2012). The individual self corresponds most closely to the independent self, as defined by Markus and Kitayama (1991). The relational self is at the interpersonal level and is the self-concept derived from connections and role relationships with significant others. The collective self is at the group level, which corresponds to the concept of social identity, as represented in social identity theory and self-categorization theory (Hogg and Abrams, 1988; Sedikides et al., 2011). Research has indeed indicated that relational self and collective self can be distinguished. For example, Oyserman et al.’s (2002) meta-analysis of individualism and collectivism showed that European Americans were not less collectivistic than Japanese or Koreans. When researchers use a sense of belonging to in-groups and seeking others’ advice to assess collectivism, Americans rate themselves as relatively collective; when researchers use duty to in-group instead of these other ways of relating to assessing collectivism, Americans rate themselves as quite low in collectivism. They propose separating the assessment of feelings of belongingness and connectedness from the feelings of duty to the in-group. That is, they suggest assessing relationality separately from collectivism to better understand the difference between Americans and Japanese. Yuki’s (2003) framework also posits that Americans have a stronger chronic tendency to emphasize categorical distinctions between in-groups and out-groups, whereas Japanese have a stronger chronic tendency to emphasize the structure of interrelationships within groups. Furthermore, evidence from neuroscience also suggests that relational self and collective self can be distinguished. For example, a functional magnetic resonance imaging (fMRI) study (Zheng et al., 2018) found that relational-self reference, compared with collective-self reference, generated stronger medial prefrontal cortex activity among Chinese participants.

### Cultural Difference in Cognition

Nisbett et al. (2001) proposed that the Eastern cognitive system is holistic and that the cognitive systems of Western peoples are analytic. Holistic processing processes the overall message of things and tends to look at the targets and their context as a whole, focusing on the relationship between the two. Analytical processing, on the other hand, tends to process the local information of things and to separate those from their background (also see Ji and Yap, 2016 for a review). The holistic processing patterns of Asians involve a wider area of attention, whereas Westerners’ analytical processing patterns involve relatively narrow but more focused attention areas (Nisbett et al., 2001). For instance, McKone et al. (2010) found that East Asians, relative to Caucasians, showed a strong global advantage in the Navon (1977) task. Even short-term cultural priming can affect people’s eye movement: Japanese participants who watched the Japanese landscape, compared with the Japanese participants who viewed the American landscape pictures, viewed the culture-neutral images to be wider and less focused on the objects in the central area (Ueda and Komiya, 2012).

Cross-cultural studies on cognitive styles show that East Asians mainly show field dependence and that Westerners mainly show field independence (e.g., Ji et al., 2000; Kitayama et al., 2003). For example, compared with participants from individualistic cultures, participants from collectivistic cultures found it even more difficult to ignore the background information provided by the boxes, thus making more mistakes when adjusting the sticks in the rod-and-frame test (e.g., Ji et al., 2000) or in the framed-line test (Kitayama et al., 2003).

There are also cultural differences in facial holistic processing. Own-race faces are more likely to be recognized holistically, whereas other-race faces are more likely to be recognized featurally with a part-whole task (Tanaka et al., 2004). Davidoff et al. (2008) found that the Himba in north Namibian, compared to the Western observers, were more disrupted by the inversion of “Thatcherized” faces. Miyamoto et al. (2011) found that Japanese are more likely than Americans to use overall resemblance over feature-matching to identify a prototypic face, as well as that Japanese were more accurate than Americans in identifying the spatial configuration of features. Tardif et al. (2017) found that Chinese participants were tuned toward lower spatial frequencies than Canadians participants during the face recognition tasks, despite comparable low-level contrast sensitivity functions.

In addition to behavioral research, cross-cultural studies on eye movement also showed that Eastern and Western participants differ in their face processing. Eastern Asians fixed centrally on the nose region, whereas Western Caucasians primarily explored the eye and mouth regions (Blais et al., 2008; Kelly et al., 2010). Researches using the Spolight paradigm (Caldara et al., 2010) and the expanded Spotlight paradigm (Miellet et al., 2013) further confirmed that Eastern participants indeed collected more information from the nose area and that Western participants collected more information from the eyes and mouth area. Rodger et al. (2010) found similar fixation patterns for inverted faces with an upright face.

As for cross-race faces, researchers found mixed results. Blais et al. (2008) found that Eastern participants collected more information from the nose area and that Western participants collected more information from the eyes and mouth area, no matter the race of the face during the learning, recognition, and race categorization tasks. Better performance for own-race faces than for other-race faces was observed for both Eastern and Western participants. However, Fu et al. (2012) found that Chinese participants adopt different visual strategies when looking at own-race and other-race faces: In the learning or recognition stage, Chinese participants pay more attention to the central areas (the nose and the mouth) of the own-race faces and on the eyes of the other-race faces. They did not observe an own-race bias in the face recognition task. Yi et al. (2015) further confirmed the above results with Chinese participants.

### Self-Construal Priming and Cognition

Self-construal can be temporarily activated in a laboratory. Based on the classic dichotomy theory of self-construal (interdependent self and independent self) proposed by Markus and Kitayama (1991), three priming methods were commonly used to manipulate the self-construal. Two of them were developed by Trafimow et al. (1991). One is the instruction-priming task. They instructed participants to think about the differences between themselves and their family and friends for independent-self priming or to think about something they have in common with their family and friends for interdependent-self priming. The results showed that participants who received the independent self-priming indeed made more idiocentric responses and fewer group responses than participants who received interdependent self-priming in the self-attitude measure. The other is the story-reading task. The story is about a king asking a general, whom he attached great importance to, to recommend a soldier to aid the king. In the story of independent-self priming, the general recommends the soldiers based on the subsequent benefit related to himself, such as solidifying dominion and increasing prestige for himself. In the story of interdependent-self priming, the general recommends a member of his family based on the interest related to his family, such as showing his loyalty to his family and increasing the power and prestige of the family. The third method is the pronoun-circling task firstly developed by Brewer and Gardner (1996), modified by Gardner et al. (1999). In this method, participants were presented an essay and were asked to circle the pronouns (e.g., I, my, mine) communicating prime independent self or pronouns (e.g., we, our, ours) communicating prime interdependent self or control (e.g., it, mountain).

As mentioned above, the tripartite self-construal model is complementary to the dichotomy theory. Some researchers adopted a self-referential task that requires judgment of whether a trait can describe the self or others (e.g., a celebrity, mother, or father) or a group (e.g., Chinese) to activate individual self, relational self, and collective self, respectively (e.g., Mamat et al., 2014; Zheng et al., 2018). In corresponding pronoun-circling priming methods, the independent self is primed in the same way as that in the dichotomy model of self, whereas the methods to manipulate relational self and collective self are different from interdependent-self priming, mainly depending on the interpersonal contextual cues and pronouns used in the priming task. For example, Brewer and Gardner (1996) preliminary operationally distinguished the relational level of self-construal from the collective level of self-construal by using the pronoun-circling task. They instructed participants to read two stories and to circle “we, they, or it” in them. They manipulated the size of the group in these stories: one story is “A Trip to the City,” which involves a small group of people, and the other is a story about attending and watching a football game at a large stadium, which involved a large group of people. Although both relational descriptions and collective descriptions were significantly or marginally significantly greater in the *we* prime than in the *it* prime or *they* prime, collective descriptions were clearly greater in the *we* prime condition, and this effect was particularly pronounced for the lager *we* context. Kashima et al. (2011) used a similar but different pronoun-circling task to prime the tripartite self-construal. The individual-self prime story used first-person singular pronouns only (*I, me, my*). The relational narrative told the story about “my parent and I,” using first-person plural pronouns only (*we, us, our*). The collective narrative described a large group from the third-person perspective, using the third-person plural pronouns (*they, them, their*). The results showed that Asian’s social self increased in the relational prime condition, whereas Australian’s social self was prominent in the collective prime condition.

The pronoun-circling priming method has been used in a large body of studies and has been validated to affect cognitive processing, specifically the global/local preference (Kühnen and Oyserman, 2002; Lin et al., 2008; Lin and Han, 2009; Springer et al., 2012; Liu et al., 2015; Choi et al., 2016). For example, researchers found faster responses to the global than to the local targets in the Navon (1977) task (a global precedence effect) in participants exposed to the interdependent self-construal priming, but they found faster responses to the local than to the global targets (a local precedence effect) in participants with the independent self-construal priming (Kühnen and Oyserman, 2002; Lin and Han, 2009). This may be due to varied spatial attention by self-construal priming. The evidence is from participants who were primed with independent self-construal and who completed the local task with higher P1 than the global task; participants who were primed with interdependent self-construal completed the global task with higher P1 than the local task (Lin et al., 2008). P1 amplitude is modulated by spatial attention (Heinze et al., 1994; Martínez et al., 1999, 2001; Di Russo et al., 2003). Lin and Han (2009) found that the flanker compatibility effect was increased by the interdependent priming relative to the independent and control priming, indicating an increasing attention scope for interdependent priming. Liu et al. (2015) employed a focal-peripheral random-dot paradigm, and they also found that the attention scope is selectively modulated by self-construal priming. The interdependent self-construal resulted in the broadening of the attention scope, together with biased information processing in favor of the visual stimuli that share the same feature (e.g., color etc.), as the focally attended stimulus. This modulation is mainly reflected by varying the degree of suppression on the processing of the incongruent contextual stimuli that do not share visual features with the focal object. Springer et al. (2012) used superimposed face-place stimuli, and they found that independency primed participants were less affected by distractors appearing in the presence of a target (i.e., smaller interference effect) than interdependently primed participants. Choi et al. (2016) found that participants attend more to the context changes in a change-blindness task following interdependent self-construal priming than following independent self-construal priming.

### Present Study

Given the cultural differences in self-construal, the cultural difference in cognition, and the effect of self-construal priming on cognition, a reasonable question is whether the cultural difference in self-construal mirrors the cultural difference in cognition. The purpose of the present study was to tackle this issue by examining whether the cultural difference of self-construal mirrors the cultural difference in face processing.

To the best of our knowledge, only a few research (Sui and Han, 2007; Sui et al., 2013; Ng et al., 2015; Ramon et al., 2016) reported the research on self-construal and face processing. Ng et al. (2015) used the self-construal scale (Singelis, 1994) to measure participants’ interdependence and to examine its correlation with old-new face recognition accuracy. They found that European Canadians with chronic interdependent-self performed greater recognition for own-race (White) but not for other-race (East Asian) faces, whereas for East Asians, higher interdependence predicted worse recognition for both own- and other-race faces. Ramon et al. (2016) recorded the eye movements of Western Caucasian and Eastern Asian observers after inter- and independent priming, whereas they performed an old/new recognition task for same- and other-race faces. They found that self-construal priming did not determine subjects’ fixation patterns during the perceptual processing of faces, with Caucasians and Asians persistently deploying their culturally preferred visual sampling strategies. However, their results were inconsistent with those of Ng et al. (2015) and did not support the view that individual differences in self-construal account for the cultural differences in face processing. Other studies (Sui and Han, 2007; Sui et al., 2013) focused on self-face processing. Their results showed that dichotomy self-construal priming can modulate the neural responses when participants judge the orientations of own faces or familiar faces in the right middle frontal cortex (Sui and Han, 2007) and in the N2 component (Sui et al., 2013).

It should be noted that circling *we our* pronouns for priming interdependent self in Ramon et al. (2016) is actually meant to prime the collective self more than the relational self (Brewer and Gardner, 1996). Thus, whether relational priming takes effect on face recognition remains unknown. What’s more, whether self-construal priming affects holistic face processing, and race categorization also remains unknown. Therefore, it is necessary to investigate whether tripartite self-construal priming influences face recognition, holistic face processing, and race categorization.

Generally, we used the pronoun-circling paradigm (Brewer and Gardner, 1996; Gardner et al., 1999) and the story-reading task (Trafimow et al., 1991) to manipulate self-construal. These two tasks were the two commonly used methods for priming self-construal (Oyserman and Lee, 2008). Some research (see Experiment 1 in Gardner et al., 1999) found no difference between their self-construal priming effect. However, a meta-analysis of Oyserman and Lee (2008) showed a larger priming effect size of story-reading task than that of pronoun-circling task. Given that the pronoun-circling task, compared with the story-reading task, was more popular, especially in research with Chinese participants (e.g., Sui and Han, 2007; Lin et al., 2008; Lin and Han, 2009; Sui et al., 2013; Liu et al., 2015) and was easier to prime tripartite self, we mainly used pronoun-circling task in the present study, and only used story-reading task in one experiment (Experiment 3A) to examine whether the results of pronoun-circling priming could be generalized with the story-reading priming.

As for the pronoun-circling task, we used the well-validated and popular pronoun-circling paradigm of the dichotomy self-construal model (e.g., Kühnen and Oyserman, 2002; Sui and Han, 2007; Lin et al., 2008; Lin and Han, 2009; Springer et al., 2012; Sui et al., 2013; Liu et al., 2015; Choi et al., 2016) in some experiments. Participants should read one story with plural pronouns (e.g., we, our) for interdependent-self priming, one with singular pronouns (e.g., I, my) for independent-self priming, and one with impersonal pronouns (e.g., it, its) in the control group. In some experiments, we used an adapted tripartite pronoun-circling paradigm based on previous studies (Brewer and Gardner, 1996; Kashima et al., 2011; Mamat et al., 2014; Zheng et al., 2018). Similarly, a story describing a trip was used. The pronoun-circling stories to prime independent self, collective/interdependent self, and the control condition were used in the same way as those in the dichotomy self-construal priming method. However, the story used to prime the relational self was written from the perspective of “my parents and I,” involving only a small group of people, using the relational pronouns *my parents and/or I*.

In the present study, we first asked whether self-construal priming affects holistic face processing in Experiment 1 and Experiment 2. We used the featural-spacing change task (Mondloch et al., 2002; Miyamoto et al., 2011) in Experiment 1A, as well as the standard complete composite face task (e.g., Zhou et al., 2012; or see Richler and Gauthier, 2014 for a review) in Experiment 1B to examine holistic processing. Both Experiments 1A and 1B used the dichotomy pronoun-circling task to prime self-construal. Experiment 2 used a revised composite face task (Richler et al., 2014) to measure holistic processing and a tripartite pronoun-circling task to prime self-construal.

Previous studies have shown that after priming the independent self, the participants are more inclined to analytical processing, and after priming the interdependent self, they tend toward holistic processing (Kühnen and Oyserman, 2002; Lin and Han, 2009; Springer et al., 2012; Liu et al., 2015; Choi et al., 2016). Therefore, we hypothesized that Chinese participants who were primed with interdependent/collective or relational self, compared with those who were primed with independent self, were more sensitive to configural features or were more likely to participate in holistic processing.

However, it should be noted that the definitions of holistic processing varied in different paradigms, and there was no correlation among these different types of holistic face processing, which implied that facial holistic processing may reflect distinct facial perceptual mechanisms (Wang et al., 2012; Rezlescu et al., 2017). Therefore, we adopted various paradigms of holistic face processing to examine which paradigm(s) is(are) sensitive to self-construal priming, and whether the effect of self-construal priming on holistic face processing could be generalized to various paradigms.

Second, we investigated whether self-construal priming affects face recognition. We used only own-race faces and used the Chinese version of the Cambridge Face Memory Test (CFMT-Chinese; McKone et al., 2012) to measure face recognition in Experiment 2, used own-race faces and other-race faces and used old-new face recognition task to measure face recognition in Experiment 3A and Experiment 3B, and recorded eye movement in Experiment 3B. Experiment 3A used an adapted Chinese version of the story-reading task (Trafimow et al., 1991) to prime self-construal. Experiment 3B adopted a tripartite pronoun-circling task to prime self-construal. Own-race faces are more likely to be recognized holistically, whereas other-race faces are more likely to be recognized featurally with a part-whole task (Tanaka et al., 2004; however, see Hayward et al., 2013 for a review for different results with other holistic tasks in Hayward et al., 2008; Wiese et al., 2009; Mondloch et al., 2010; Horry et al., 2015), and individual differences in holistic processing could predict face recognition (Richler et al., 2011; Wang et al., 2012; DeGutis et al., 2013; however, see different results in Konar et al., 2010; and mixed results in Rezlescu et al., 2017). Therefore, interdependent/collective or relational priming would be expected to increase own-race face recognition performance; independent priming would be expected to enhance other-race face recognition.

It should be noted that the measures of face recognition in those research also varied: four/ten-alternative forced choice identification task in some research (Konar et al., 2010; Richler et al., 2011), old-new face recognition task in some research (Wang et al., 2012), and the Cambridge Face Memory Test (Duchaine and Nakayama, 2006) in other research (Richler et al., 2011; DeGutis et al., 2013; Rezlescu et al., 2017). Therefore, we adopted various paradigms of face recognition to examine which paradigm(s) is(are) sensitive to self-construal priming, and whether the effect of self-construal priming on face recognition could be generalized to various paradigms.

In addition, Ng et al. (2015) found higher interdependence chronic self-construal predicted worse recognition for both own- and other-race faces for East Asians. What’s more, self-construal priming did not determine subjects’ fixation patterns during the perceptual processing of faces, with Caucasians and Asians persistently deploying their culturally preferred visual sampling strategies (Ramon et al., 2016). Therefore, we examine whether the results of Ramon et al. (2016) could be confirmed with Chinese participants.

Finally, we are interested in whether self-construal priming affects race categorization in behavior performance and eye movement in Experiment 3B. Race categorization is different from face recognition in that face recognition task showed an own-race advantage, while race categorization showed an other-race advantage (Levin, 2000; Ge et al., 2009). Blais et al. (2008) showed Caucasians and Asians persistently deploying their culturally preferred visual sampling strategies in the face learning, face recognition, and race categorization stages. It has been shown that self-construal priming did not influence the eye movement patterns during the face learning and face recognition stages (Ramon et al., 2016). However, whether self-construal priming affects race categorization remains unknown. According to Blais et al. (2008), we expect to observe different eye movement patterns for interdependent/collective or relational priming and for independent priming with the former similar with Asians’ pattern and the latter similar with Caucasians’ pattern. However, according to Ramon et al. (2016), it is also possible that self-construal priming would not affect race categorization, similar with the result of face learning and that of face recognition.

The current research has been approved by the Institutional Review Board of the Psychology Department of Sun Yat-sen University. Each participant in each experiment signed a consent form before taking part in the experiment.

## Experiment 1: Self-Construal and Holistic Face Processing

In this experiment, we are interested in whether self-construal priming affects holistic face processing. We used the featural-spacing change task in Experiment 1A, as well as the standard complete composite face task in Experiment 1B to measure holistic processing. Both Experiments 1A and 1B used the dichotomy pronoun-circling task to prime self-construal.

If the cultural difference of self-construal mirrored the cultural difference in holistic face processing, the participants who were primed with interdependent/collective or relational self, compared with those who were primed with independent self, would be more likely to participate in holistic processing.

### Experiment 1A: Space and Feature

#### Method

##### Participants

One hundred and nine students (Mean age = 18.84 years, SD = 1.21; 47 males, 62 females) of Sun Yat-sen University participated in this experiment for payment and were randomly assigned to three priming groups. Three subjects were excluded from the final analysis because they ran the wrong program that did not correspond to the priming group they were assigned to. The final data included 35 in the control group, 34 in the independent group, and 37 in the interdependent group.

##### Materials

**Self-construal priming**: We manipulated the self-construal level by employing the pronoun-circling task (Gardner et al., 1999). Because we used two tasks of face processing in the current experiment, we used two Chinese essays describing a trip in the priming procedure. Each essay converted into three versions that contained independent (e.g., I, my), interdependent (e.g., we, our), and control (e.g., it, its) pronouns, with the same number of pronouns to circle.

**Figure 1:**
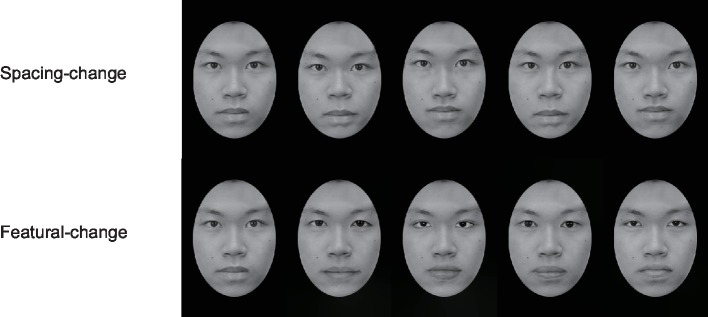
Example of an original face (the left-most face in each row), his spacing-composite faces (top row), and his featural-composite faces (bottom row) used in Experiment 1A. These illustrated examples did not appear in the actual experiment. Written informed consent was obtained from the individual for the publication of this image.

**Face stimuli**: Four original Chinese faces (half male and half female) were used to generate four sets of spacing-change faces and featural-change faces. In all faces, the non-facial features such as hair, ears, and neck were removed, and the faces were generated into elliptical grayscale images of 284 × 396 pixels. As shown in Figure 1, each original face was generated into four images with changing facial spaces and four images with changing facial features. Following the previous study (Mondloch et al., 2002; Miyamoto et al., 2011), the four faces in the spacing set were created by moving the eyes in the original face down and up, shortening or widening them 4 mm (10 pixels), and moving the mouth down or up 2 mm (5 pixels) (moving eyes and mouth down; moving eyes and mouth up; moving the eyes closer together; moving the eyes farther apart). The four faces in the featural-changing set were created by replacing the eyes and mouth in the original face with the features of other same-sex faces. The replaced eyes and mouth are approximately equal in length with the eyes and mouth of the original face in order to maintain the original space position constant. In total, 36 images (4 original faces +32 generated faces) were created as stimuli.

##### Procedure

The experiment was programmed with E-prime1.1 and was run *via* a 23-inch computer with a resolution of 1,920 × 1,080 pixels. Throughout the experiment, the distance between the participants’ eyes and the computer screen was about 50 cm.

Participants were randomly assigned to a priming group. The spacing and featural-face processing procedure followed that of the previous study (Mondloch et al., 2002; Miyamoto et al., 2011). Spacing-change trials and featural-change trials were blocked to encourage participants to use specific face-processing strategies. The order of the two blocks was balanced across participants.

In each block, the instructions informed the following: “These faces all look alike, but they are all different people. Two faces flash up fast on the screen successively several times. The task is to judge whether the two faces are the same or different as accurately and as quickly as possible.” As an illustration, a Chinese male face and its four modified faces with spacing/featural change were presented on the screen. Then, participants were given eight practice trials: one same and one different trial for each face set. After that, participants read the printed priming stories and circled the pronouns. Then they completed 160 formal trials, with half being same trials and the other half being different trials.

In each practice trial, first there was a 600-ms inter-trial blank interval, and then the first face was displayed 360 ms, followed by a 100-ms blank, and then the second face was displayed until participants responded by pressing “1” (same) or “2” (difference), followed by a 1,500-ms feedback. The accuracy rate of practice trials should reach 60% to start the formal experiment.

In each formal trial, first there was a 600-ms inter-trial blank interval, and then the first face was displayed 200 ms at the center of the screen. After a 300-ms blank interval, the second face appeared until the participants’ response.

Each face served as the first face as often as the second face, and each face was presented on the same trials as often as the different trials.

At the end of the experiment, participants were asked to complete a questionnaire to evaluate their attention to the information on the face during the experiment on a 7-point Likert scale from 1 (completely unnoticed) to 7 (completely noticed). The questionnaire contains gazing ratings of two types of information on the face: one is featural information such as nose, eyebrows, eyes, and mouth; and the other is integral information such as expression, overall impression, and configuration (Miyamoto et al., 2011).

#### Results and Discussion

The analysis of the questionnaire of gazing ratings showed no significant effect (*p*s > 0.35).

Miyamoto et al. (2011) showed that Japanese are more likely than Americans to use overall resemblance over feature-matching to identify a prototypic face, as well as that Japanese were more accurate than Americans in identifying the spatial configuration of features. Therefore, if the cultural difference of self-construal mirrors the cultural difference in face processing, the participants who were primed with interdependent/collective or relational self, compared with those who were primed with independent self, would be more sensitive to spacing-change.

**Figure 2:**
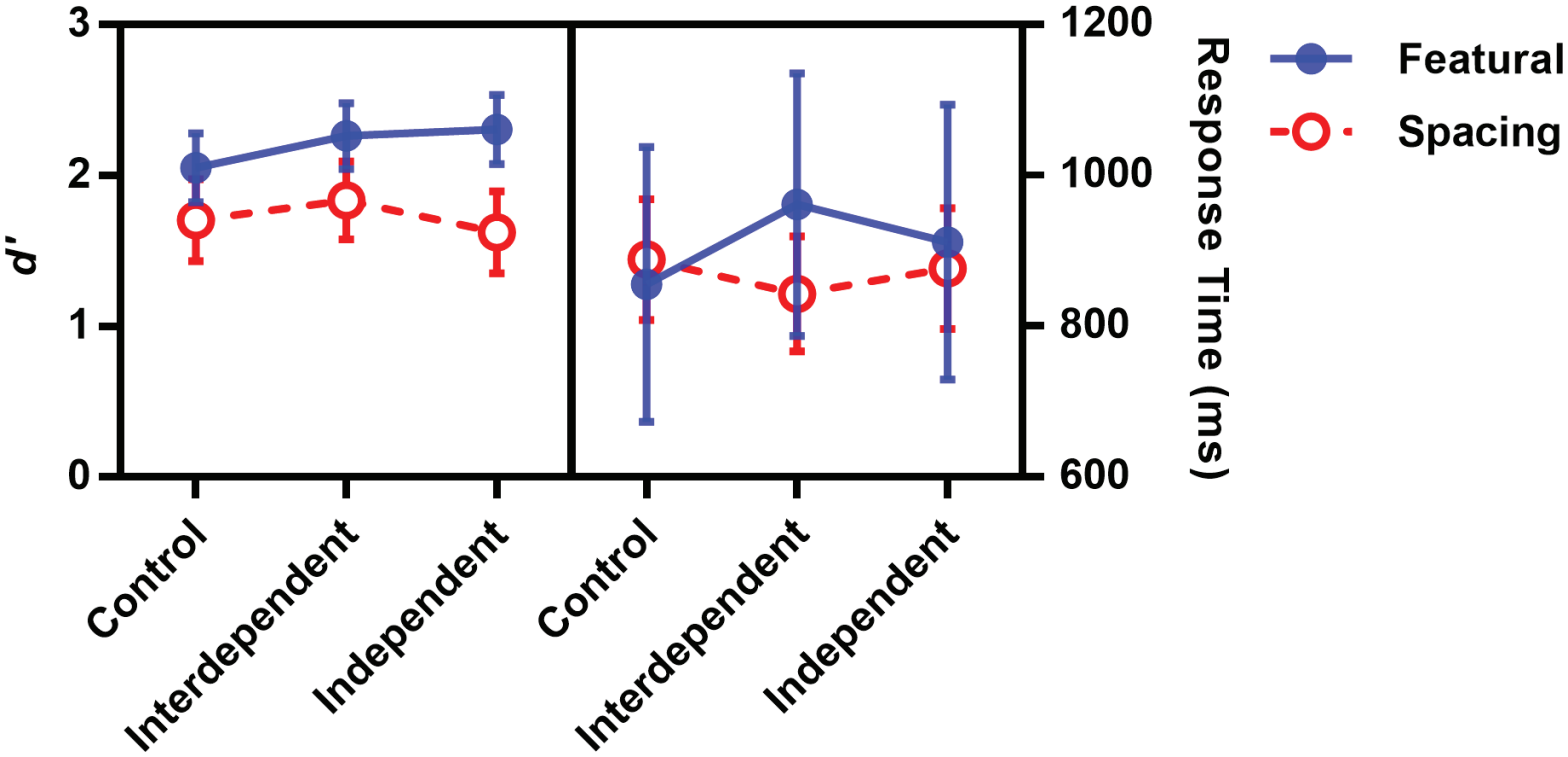
Mean sensitivity and correct response time as a function of priming and feature change type in Experiment 1A. The error bar included here and elsewhere refers to a 95% confidence interval.

Sensitivity *d*′ [Z(Hit)-Z(False Alarm)] of each participant was submitted to a 3 (priming: control, interdependent, independent) × 2 (feature type: featural, spacing), mixed-design ANOVA. Four participants’ *d*′ was below two standard deviations from the mean in either the featural task or the spacing task, so their data were discarded. Results (see Figure 2) showed a significant main effect on the feature type, *F*(1, 99) = 38.94, *p* < 0.001, $\eta_{p}^{2}$ = 0.28, with better performance for featural change than for spacing change. The main effect of priming and its interaction with feature type did not reach significance, *p*s > 0.19, indicating that self-construal priming did not affect participants’ sensitivity to spacing change or featural change.

The analysis of response time on the correct trials showed no significant effect (*p*s > 0.37), further confirmed that self-construal priming did not affect participants’ sensitivity to spacing change or featural change (Figure 2).

### Experiment 1B: Standard Composite Effect

#### Method

##### Participants

Twenty-nine Sun Yat-sen students (mean age = 19.93 years, SD = 1.71; 16 males, 13 females) participated in this experiment for payment. All participants were with normal or corrected normal vision.

##### Materials

**Self-construal priming**: In this experiment, the self-construal levels were manipulated within the subjects. Same as Experiment 1A, we also used the pronoun-circling task (Gardner et al., 1999), but we asked the participants to circle the neutral words (e.g., mountain, see Liu et al., 2015) here instead of impersonal pronouns (e.g., it, its) in the control condition.

**Face stimuli**: Eight Chinese male faces were used to generate stimuli by removing the hair, ear, neck, and other non-facial features from facial images and were processed into grayscale images of 180 × 240 pixels.

##### Procedure

The experiment was programmed by E-prime1.1 software. The stimuli were presented on a 21-inch computer screen with a resolution of 1,024 × 768 pixels.

The composite face task consists of a training block and three formal blocks. The training block consisted of eight practice trials with feedbacks, and then participants were required to complete the pronoun-circling task before each formal block. The inter-block intervals lasted 1 min. The order of three priming tasks was counterbalanced across participants.

**Figure 3:**
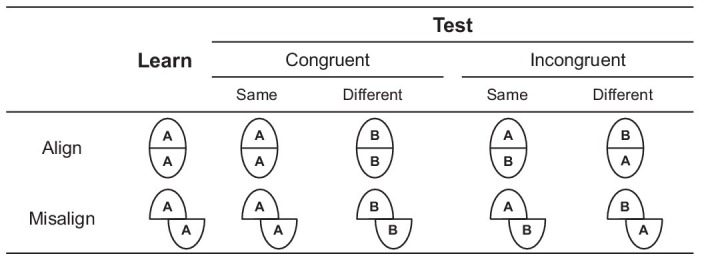
Illustration of the standard composite task in Experiment 1B. Participants’ task was to determine whether the tops of the learn- and test-composite face were the same or different. In the congruent trials, the tops and bottoms were both the same or different; in incongruent trials, the tops were the same and the bottoms were different, or vice versa.

The trial procedure of the composite face task followed that of Zhou et al. (2012). In each trial, a 200-ms fixation was displayed at the center of the screen, followed by a 250-ms blank screen. The first composite face (the learn face in Figure 3) was displayed for 200 ms, followed by a 500-ms blank screen, and the second composite face (the test face in Figure 3) was displayed for 2,000 ms or until participants responded. Participants were required to determine whether the tops of the two composite faces were the same or different as quickly and accurately as possible. Half of the trials were the same correct response, and the other half were the different correct response. In congruent trials, the tops and bottoms were both the same or different; in incongruent trials, the tops were the same and the bottoms were different, or vice versa. Each formal block consisted of four conditions (alignment × congruence) with 32 trials in each condition, totaling 128 trials.

#### Results and Discussion

Two participants’ data were discarded because their accuracies were below two standard deviations from the mean (mean = 0.85, SD = 0.06).

**Figure 4:**
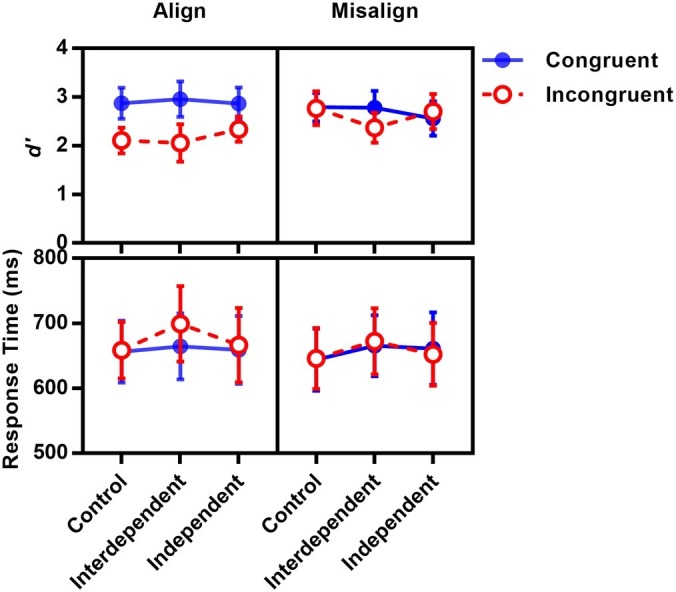
Mean sensitivity and correct response time as a function of priming, congruency, and alignment in Experiment 1B.

Sensitivity *d*′ (see Figure 4) of each participant was submitted to a 3 (priming: control, interdependent, independent) × 2 (congruency: congruent, incongruent) × 2 (alignment: aligned, misaligned) repeated ANOVA. If the cultural difference of self-construal mirrored the cultural difference in face processing, the participants who were primed with interdependent/collective or relational self, compared with those who were primed with independent self, would show more holistic face processing (i.e., larger alignment × congruence effect).

There was a significant main effect in congruency [*F*(1, 26) = 27.88, *p* < 0.001, $\eta_{p}^{2}$ = 0.52] and a trend toward an alignment effect [*F*(1, 26) = 2.92, *p* = 0.10, $\eta_{p}^{2}$ = 0.10]. Their interaction reached significance, *F*(1, 26) = 18.48, *p* < 0.001, $\eta_{p}^{2}$ = 0.42, indicating a holistic face processing. Further simple effect analysis showed that when the top half and the bottom half of a face were aligned, the bottom half indeed impacts the processing of the top half, with a better performance in congruent condition than that in incongruent condition, *p* < 0.001. In the misaligned condition, however, no difference between congruence and incongruence was observed, *p* = 0.32.

Importantly, a significant interaction between priming and congruency was observed, *F*(2, 52) = 3.33, *p* = 0.04, $\eta_{p}^{2}$ = 0.11. A simple effect test showed no congruency effect for the independent condition (*p* = 0.90), whereas it showed a congruency effect for the control condition (*p* < 0.01) and interdependent condition (*p* < 0.001). Other main effects or interactions did not reach significance (*p*s > 0.30).

Although priming did not affect congruency × alignment holistic processing, priming influenced the congruency effect with the same pattern mirroring the cultural difference in holistic processing.

The correct mean response time (see Figure 4) of each participant was submitted to a 3 × 2 × 2 repeated ANOVA. There was a marginal significant main effect of congruency [*F*(1, 26) = 3.07, *p* = 0.09, $\eta_{p}^{2}$ = 0.11] and alignment [*F*(1, 26) = 3.52, *p* = 0.07, $\eta_{p}^{2}$ = 0.12]. Their interaction reached significance, *F*(1, 26) = 5.67, *p* = 0.03, $\eta_{p}^{2}$ = 0.18. Importantly, we observed a significant interaction between priming and congruency, *F*(1, 26) = 3.44, *p* = 0.04, $\eta_{p}^{2}$ = 0.12. A simple effect test showed no congruency effect for the control condition (*p* = 0.75) and independent condition (*p* = 0.90), whereas it showed a congruency effect for the interdependent condition (*p* = 0.005). Other main effects or interactions did not reach significance (*p*s > 0.35).

Similar to the result of *d*′, the result of the response time showed that priming could influence the congruency effect. However, compared with the control condition, the interdependent priming only affects response speed, so as to enlarge the congruency effect, whereas the independent priming only affects sensitivity, so as to shrink the congruency effect.

## Experiment 2: Self-Construal, Revised Composite Effect, and Face Recognition

Given that there is low reliability (around 0.2) for the standard composite face task (DeGutis et al., 2013; Ross et al., 2015), Richler et al. (2014) proposed the Vanderbilt holistic face processing test (VHFPT) as a revised composite effect task. VHFPT has good reliability, around 0.4–0.7 (Richler et al., 2014; Wang et al., 2016). In addition, according to the tripartite model of self-construal (Brewer and Gardner, 1996), self-construction can be subdivided into independent/individual self, relational self, and collective self. Therefore, in this experiment, we used VHFPF to measure holistic face processing and used the pronoun-circling task to prime the independent, relational, and collective selves to examine whether the self-construal priming effect on holistic face processing could be generalized. We were also interested in whether self-construal priming would affect face recognition.

Own-race faces are more likely to be recognized holistically, whereas other-race faces are more likely to be recognized featurally (Tanaka et al., 2004), Chinese participants were tuned toward lower spatial frequencies than Canadians participants during the face recognition tasks (Tardif et al., 2017), and individual differences in holistic processing could predict face recognition (Richler et al., 2011; Wang et al., 2012; DeGutis et al., 2013). Therefore, if the cultural difference in self-construal mirrored the cultural difference in face holistic processing and face recognition, the participants who were primed with interdependent/collective or relational self, compared with those who were primed with independent self, would show more holistic face processing and better face recognition performance.

### Method

#### Participants

Eighty-six undergraduates (mean age = 19.66 years, SD = 1.25; 39 males, 47 females) of Sun Yat-sen University participated in the study. All participants had normal or corrected normal vision and were randomly assigned to four groups (about 21 or 22 participants in each group).

#### Materials

**Self-construal priming**: Based on the tripartite self-construal model (Brewer and Gardner, 1996), we used an adapted pronoun-circling task to prime independent, relational, and collective selves. The collective-self priming method was the same as that used for interdependent-self priming in Experiment 1. The relational self was manipulated by reading the story with pronouns of *my parents and me, parents,* or *me* and circling those pronouns in it. Independent-self priming and control conditions were identical to those in Experiment 1A.

**Figure 5:**
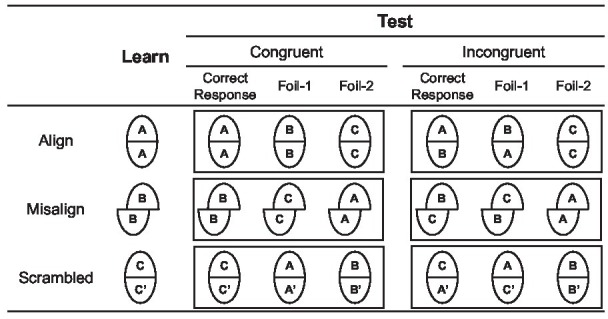
Illustration of six trials in the revised composite task in Experiment 2. Participants’ task was to select one composite face with the same target parts (here, the target is the top half) as that of learning composite face from three test composite faces (each rectangle above). Apostrophes indicate that distractor parts of the composite faces (bottom parts in this figure) have been phase-scrambled.

**Figure 6:**
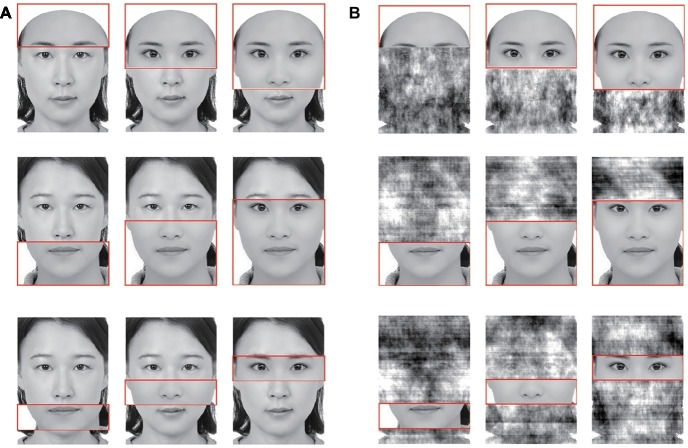
Examples of nine different kinds of target parts in alignment condition **(A)** and scramble condition **(B)** of the VHFPT in Experiment 2. In all trials, target parts were identified by a 2-pixel wide red line box. These illustrated examples did not appear in the actual experiment. Written informed consents were obtained from the two individuals for the publication of these images.

**Faces in VHFPT**: Six female Chinese celebrity faces obtained from the Internet and 216 unfamiliar Chinese faces (108 males and 108 females) were used as stimuli. They were converted to 86 × 120 pixels grayscale images. The celebrity-face set was used for practice. The unfamiliar faces were grouped into 36 sets of six same-sex faces. Within each set, three faces were used to generate the target parts, and the other three faces were used to generate the distractor parts. Each set of faces was combined into 3 alignments (aligned, misaligned, and scrambled) × 2 congruencies (congruent or incongruent) for a total of six trials per set (see Figure 5). Unfamiliar face sets were randomly assigned to nine target part conditions (see Figure 6): top and bottom third (86 × 40 pixels); top half and bottom half (86 × 60 pixels); top and bottom two-thirds (86 × 80 pixels); and eyes, nose, and mouth (86 × 23 pixels). The target parts in the learning and test phases were marked with a red line with a width of two pixels. The top portions of targets were cropped to remove hair to avoid salient non-face cues. Each target part condition contained two female sets and two male sets.

Each target face was the correct answer for one alignment condition pair (congruent and incongruent trials) and served as a foil in the other two alignment conditions. The composite face in the learning phase was created by pairing one of the three target parts and distractor parts in one set. The same composite face was used for congruent and incongruent trials within each alignment condition. In the test phase, three composite faces (correct response, foil 1, and foil 2) were displayed simultaneously. In congruent trials, the target part of the correct-response face was paired with the same distractor part of the composite face in the learning phase. The other two foil faces were composited with the remaining two target and distractor parts. In incongruent trials, the target part of the correct-response face was paired with the distractor part of foil 1, and the learning composite distractor part was paired with the target part of foil 1. The second foil face in congruent trials was the same as that in incongruent trials.

In misaligned condition, the target and distractor parts were aligned in study composites and were misaligned in test composites. The extent of misalignment of the two parts was constant in the experiment at 20.5 pixels to the right of the interfering part. In scrambled condition, the distractor parts were degraded using the random image structure evolution algorithm proposed by Sadr and Sinha (2004) and refer to the 65% picture distortion rate used in Richler et al.’s study (2014).

**Face recognition**: We used the Chinese version of the Cambridge face memory test (CFMT-Chinese; McKone et al., 2012) to measure face recognition. The procedure of CFMT-Chinese followed the same procedure of the CFMT (Duchaine and Nakayama, 2006). The Chinese male face stimuli were developed in the same way as those in CFMT.

#### Procedure

The VHFPT, running with E-prime 1.1 software, and CFMT-Chinese, running with MATLAB software, were displayed on a monitor with a resolution of 1,024 × 768 pixels. The distance between the participants and the screen was 65 cm.

Participants were randomly assigned to one of four priming groups. Each group accepted one type of self-construal priming with the pronoun-circling priming task. Participants then immediately completed the VHFPT (Richler et al., 2014), followed by the CFMT-Chinese (McKone et al., 2012).

**Vanderbilt holistic face processing test (VHFPT)**: In each trial, one composite face (the learn face in Figure 5, target part was outlined in red as shown in Figure 6) was presented for 1,000 ms, followed by a 500-ms mask. Then, a set of three test faces (the test faces in Figure 5, target parts were outlined in red) appeared until participants made a response. The masked pictures were black and white random dots and were of the same size as the composite faces (86 × 120 pixels). The task was to judge which of the three composite faces contained the same target part as the study composite. The response keys (J, K, and L) appeared below each test image. The position of correct faces was randomized.

Trials were blocked by target parts. The order of nine blocks was randomized among participants. Each block started with six practice trials generated by the celebrity face set, followed by 24 formal trials generated by four face sets. In order to ensure that at least two trials separated trials generated from the same face set, trials were presented in preset random order. The VHFPT contains 270 trials (54 practice trials and 216 formal trials).

**Cambridge face memory test (CFMT-Chinese)**: The CFMT-Chinese (McKone et al., 2012) was presented using the standard procedure in Duchaine and Nakayama (2006), consisting of four phases (practice, same images, novel images, and novel images with noise). The practice phase familiarizes participants with the procedure used in the “same images” phase by presenting carton faces in the same fashion that target faces will be presented. In the “same images” phase (18 trials), there were six different male faces presented from three viewpoints each (a left 1/3 profile, a frontal view and a right 1/3 profile). In each trial, a face was displayed for 3 s, then three test items consisting of one target face and two distractor faces were presented. Participants were instructed to choose the individual who they were just shown. In this phase, the target face in study and test are the same image. In the subsequent “novel images” phase, six target faces with a frontal view were displayed for 20 s. Then, 30 trials of three-alternative forced-choice test items were displayed in a fixed random order. Participants were asked to select one of the six target faces from three test faces. The target images in the test items were novel images that varied in pose, lighting, or both. The procedure in the “novel images with noise” phase was similar, except that there were 24 trials in this phase, and the test faces were all novel images with Gaussian noise.

### Results and Discussion

Four participants’ accuracies were below two standard deviations from the mean (Mean = 0.86, SD = 0.06), so their data were discarded.

Compared to traditional face composite effect tasks that measure the holistic processing effect of faces by the interaction effect of congruency (congruent or incongruent) × alignment (aligned or misaligned), VHFPT adds a new set of scramble conditions. The holistic processing effect in the present experiment was measured as the congruency (congruent or incongruent) × alignment (aligned, misaligned, or scrambled) interaction.

#### Self-Construal Priming and the Holistic Processing Effect

**Figure 7:**
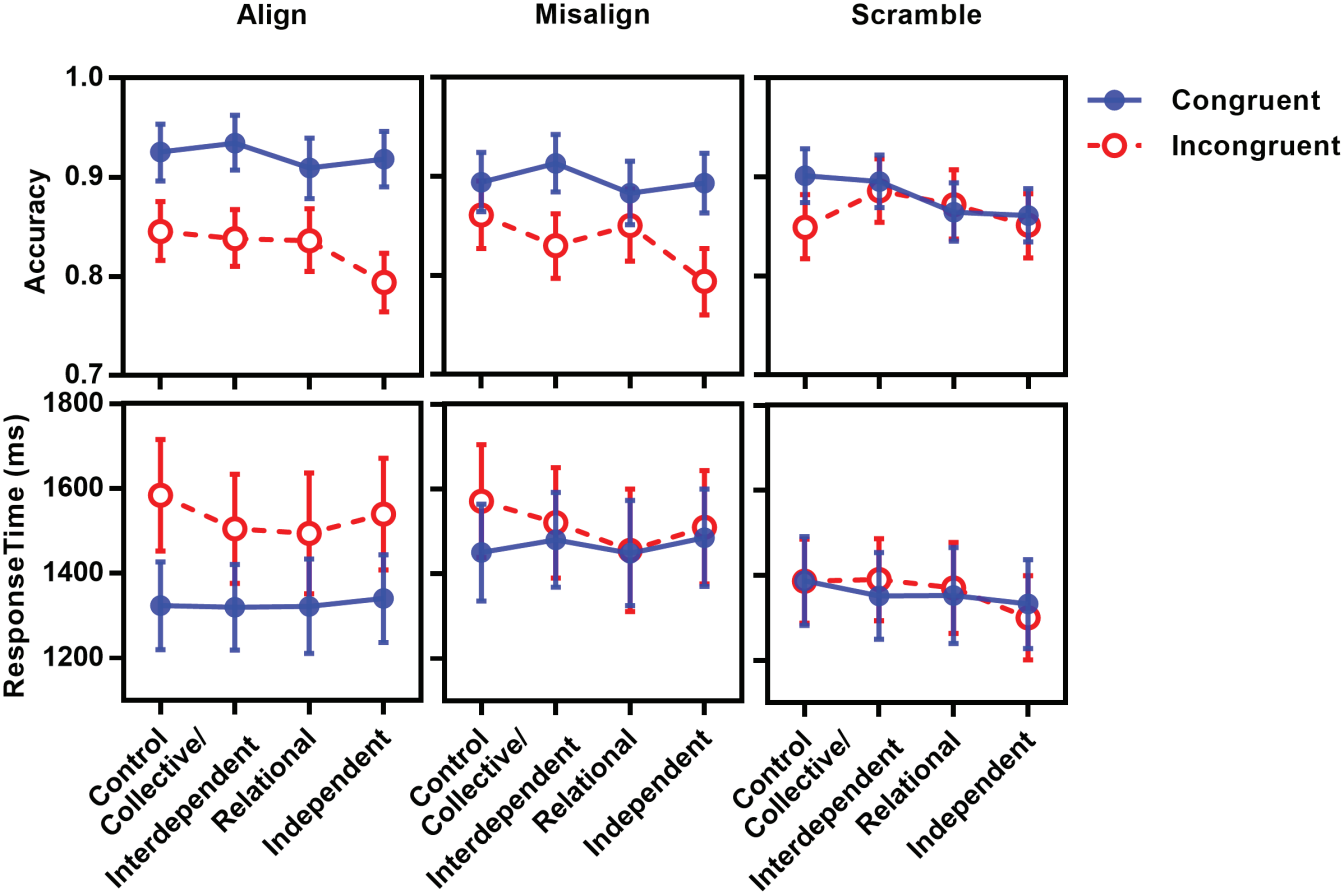
Accuracy (top panel) and correct response time (bottom panel) as functions of priming, congruency, and alignment in Experiment 2.

The accuracies of the remaining participants were submitted to a 4 (priming: control, collective/interdependent, relational, independent) × 2 (congruency: congruent, incongruent) × 3 (alignment: align, misalign, scramble) mixed-design repeated measure ANOVA. The results (see Figure 7) showed a significant main effect of congruency, *F*(1,78) = 115.84, *p* < 0.001, $\eta_{p}^{2}$ = 0.60, and its interaction with alignment, *F*(2,156) = 24.84, *p* < 0.001, $\eta_{p}^{2}$ = 0.24. Importantly, these effects were modulated by priming. Both the interaction of congruency and priming [*F*(3,78) = 3.06, *p = 0*.03, $\eta_{p}^{2}$ = 0.11] and the three-way interaction [*F*(6,156) = 2.69, *p* = 0.02, $\eta_{p}^{2}$ = 0.09] reached statistical significance. Other main effects or interactions were not observed (*p*s > 0.14).

Separate analysis for each priming group showed significant main effects of congruency for all groups (*p*s < 0.01). Significant congruency × alignment interactions were observed for all groups (*p*s < 0.02) except the control group (*p* = 0.16).

To compare the congruency effect of each priming group, we calculated the size of congruency effect (control: 0.05, relational: 0.03, collective/interdependent: 0.06, independent: 0.08) and submitted it to a one-way ANOVA. Bonferroni’s *post hoc* tests showed that only the independent group and the relational group showed significant differences, with a larger congruency effect for the independent group than that of the relational group (*p* < 0.03); the differences between other groups did not reach significance (*p*s > 0.29). That is, although we replicated the result that the congruency effect was modulated by priming in Experiment 1B, the priming pattern was different from that of Experiment 1B. The larger congruency effect in independent priming than in relational priming is inconsistent with the cultural difference in holistic processing.

To examine whether the three-way interaction of priming × congruency × alignment was due to null congruency × alignment interaction for the control group, we did a 3 (priming: collective/interdependent, relational, independent) × 2 (congruency: congruent, incongruent) × 3 (alignment: align, misalign, scramble) mixed-design repeated measure ANOVA. The three-way interaction of priming × congruency × alignment did not reach statistical significance, *F*(4,124) = 0.48, *p = 0*.75, $\eta_{p}^{2}$ = 0.02.

The 4 × 2 × 3 mixed-design repeated measure ANOVA of the correct response time of each participant showed a significant main effect of congruency; *F*(1,78) = 49.51, *p* < 0.001, $\eta_{p}^{2}$ = 0.39; alignment, *F*(2,156) = 37.21, *p* < 0.001, $\eta_{p}^{2}$ = 0.32; and their interaction, *F*(2,156) = 41.83, *p* < 0.001, $\eta_{p}^{2}$ = 0.35. No other main effect or interaction was observed (*p*s > 0.23). Differently from Experiment 1B, priming did not affect the congruency effect in RT.

#### Self-Construal Priming and Face Recognition

Two participants’ data were discarded because their CMFT performance was beyond two deviations of the mean. The CMFT percentage was submitted to a one-way ANOVA. The results showed no effect of self-construal priming on CMFT performance, *F*(3,84) = 1.31, *p* = 0.28, $\eta_{p}^{2}$ = 0.05. This result also indicated that self-construal had no effect on face recognition, consistent with Ramon et al. (2016), inconsistent with Ng et al. (2015). The null effect of self-construal on face recognition may be due to a not strong enough priming effect of pronoun-circling task. Therefore, we further examined the effect of self-construal on face recognition with story-reading task in Experiment 3.

The Pearson correlation analysis showed that the accuracy of CFMT was not correlated with the holistic processing effect neither in accuracy nor in RT no matter take scramble as baseline or misalign as baseline, *p*s > 0.26, consistent with some research (Konar et al., 2010) while inconsistent with others (Richler et al., 2011; Wang et al., 2012; DeGutis et al., 2013).

## Experiment 3: Self-Construal and Own-Race Bias

Although Experiment 2 did not show self-construal priming effect on face recognition, the purpose of Experiment 3 was to investigate whether the result could be replicated with story-reading priming task (Experiment 3A), whether the result could be generalized to other race face recognition and race categorization task (Experiment 3A and 3B), and how self-construal would affect the eye movement during face learning, face recognition, and race categorization (Experiment 3B).

### Experiment 3A: Self-Construal, Own-Race Bias

#### Method

One hundred and three students of Sun Yat-sen University (mean age = 18.65, SD = 0.79; 41 males, 62 females) participated in this experiment. All participants had normal or corrected normal vision and were randomly assigned to three groups. Each group received one type of self-construal priming and then completed a face recognition task immediately. The face recognition task was programmed with E-prime1.1 and done on a computer with a resolution of 1,280 × 768 pixels.

**Self-construal priming**: In this experiment, we manipulated the self-construal level using the story-reading task in Trafimow et al. (1991). Because all participants are Chinese, we changed the backgrounds and names of the characters in those stories into Chinese style. In the control condition, no priming was done.

**Face recognition task**: The own-race faces were 20 faces photographed by our lab [some of them used in Zhou et al. (2015) and the other-race faces were 20 Caucasian faces by Minear and Park (2004)], 10 female faces and 10 male faces. We removed the non-facial features such as hair, ears, and necks, and generated the faces into elliptical grayscale images of 120 × 170 pixels.

The face recognition task consisted of two blocks, one for own-race faces and the other for other-race faces. The order of the two blocks was counterbalanced across participants. Each block consisted of a learning stage and a recognition stage. In the learning stage, 10 faces were randomly presented in turn. Each picture was presented at the center of the screen for 3,000 ms after a 500-ms fixation. Then, participants would relearn the 10 faces in the same order. Participants were asked to try to remember those faces. In the following stage of recognition, 20 pictures were randomly presented on the screen one by one. Half of the face pictures were from the learning stage and half were new faces. Participants were asked to judge as accurately as possible whether each face picture had been shown in the learning stage, pressing the “F” key for “old face” and the “J” key for “new face.”

#### Results and Discussion

Five participants’ data were discarded because their mean accuracy was beyond two standard deviations of the mean.

Sensitivity *d*′ were submitted to a 3 (priming: control, interdependent, independent) × 2 (facial race: own-race, other-race) mixed-design repeated measures ANOVA. If the cultural difference of self-construal mirrored the cultural difference of face recognition, an interdependent/collective priming would be expected to increase own-race face recognition, and independent priming would increase other-race face recognition.

Neither main effect nor interaction of *d*′ reached statistical significance (*p*s > 0.13).

Analysis of hit rates also showed no significant effect (*p*s > 0.25).

However, analysis of false alarms showed an own-race bias effect, *F*(1,95) = 16.20, *p* < 0.001, $\eta_{p}^{2}$ = 0.15. The main effect of priming and its interaction did not reach statistical significance (*p*s > 0.50).

These results indicated no priming effect of self-construal on face recognition, consistent with the results of Experiment 2 and Ramon et al. (2016), inconsistent with Ng et al. (2015).

### Experiment 3B: Self-Construal, ORB, Race Categorization, and Eye Movement

#### Method

##### Participants

Eighty-three students from Sun Yat-sen University (mean age = 19.8 years, SD = 1.59; 35 males, 48 females) participated in the experiment and were randomly assigned to four groups (22/21/20/20 for independent, relational, collective, and control groups). Three participants’ data were discarded because of failed calibration. Only 80 participants’ data were used for analysis.

##### Materials

**Self-construal priming**: We used the same pronoun-circling priming task as in Experiment 2 to manipulate self-construal level.

**Face stimuli**: A total of 40 face images (20 female faces and 20 male faces) with neutral expressions were used, including 20 Caucasian face images and 20 Chinese face pictures used in the eye movement research of Yi et al. (2015). The images were 500 × 700 pixels (11.3 × 15.8 cm) in size, viewed at a distance of 60 cm, displayed on a 17-inch screen with a resolution of 1,280 × 1,024 pixels.

**Figure 8:**
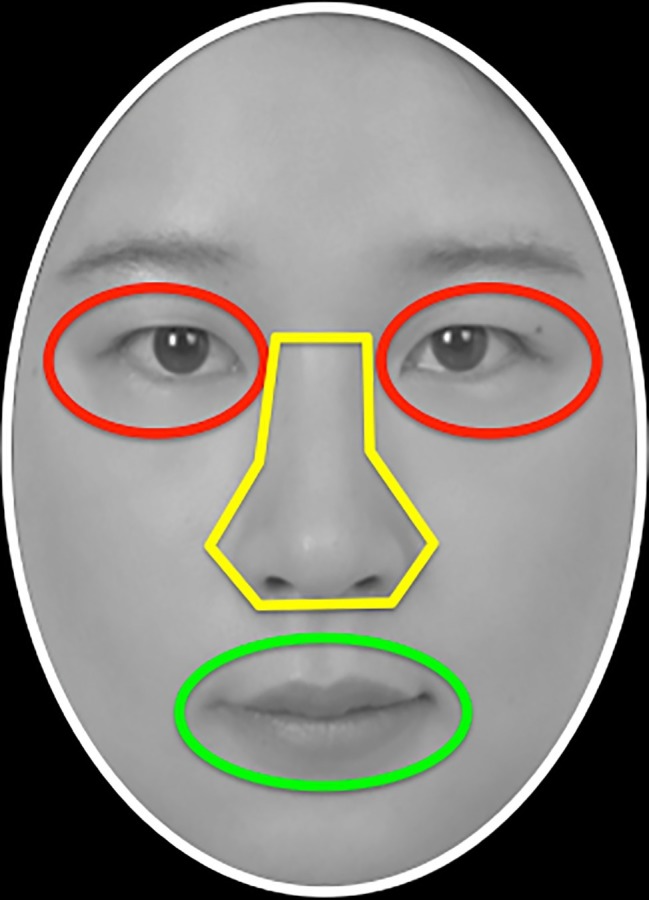
An example of face stimuli with area of interest (AOI) used in Experiment 3B. Written informed consents were obtained from the two individuals for the publication of these images. This illustrated example did not appear in the actual experiment. Written informed consent was obtained from the individual for the publication of the image.

In order to reduce the influence of irrelevant variables and to measure eye movements accurately (see Yi et al., 2015), the face images were standardized by removing the non-facial features and generating elliptical grayscale images with uniform brightness and luminance. In addition, in order to better detect the number of fixation points in the area of interest (AOI: eyes, nose, and mouth; see Figure 8), the AOIs of different faces were adjusted to be substantially the same position.

**Eye tracking**: Eye movements were recorded at a sampling rate of 1,000 Hz with the Eyelink 1000 eye tracker (SR Research), which has an average accuracy of down to 0.15° (0.25–0.5° typical), a spatial resolution of 0.01° RMS, and a gaze tracking range of 60° horizontally and 40° vertically (see EyeLink 1000 User Manual for details). Participants’ binocular movements were tracked and analyzed.

##### Procedure

The experiment was programmed and run *via* SR Research Experiment Builder software. At the beginning of the formal experiment, a 9-point randomized calibration of eye fixation was conducted. If the participant failed to complete the calibration after four attempts, he or she did not undergo the formal experiment. The calibration was then validated and repeated when necessary until the optimal calibration criterion was reached.

The following face recognition task mainly followed the procedure of Blais et al. (2008). First, participants completed a traditional face recognition task for own-race faces and other-race faces. The order of the two race blocks was counterbalanced across participants. The face recognition task was identical with that used in Experiment 3A, except that each picture was presented for 5,000 ms in the learning phase. Next, the participants completed a race categorization task. Forty faces were randomly presented at the center of the screen one by one until participants responded. Participants were required to indicate the race of the presented face and to use their dominant hands to press 1 for “Chinese” or 2 for “Caucasian.” To guarantee the self-construal priming effect, participants had to complete a pronoun-circling priming task at the beginning of the own/other face recognition task and race categorization task.

#### Results and Discussion

##### Own-Race Bias

If the cultural difference of self-construal mirrored the cultural difference of face recognition, an interdependent/collective or relational priming would be expected to increase own-race face recognition, and independent priming would increase other-race face recognition.

**Figure 9:**
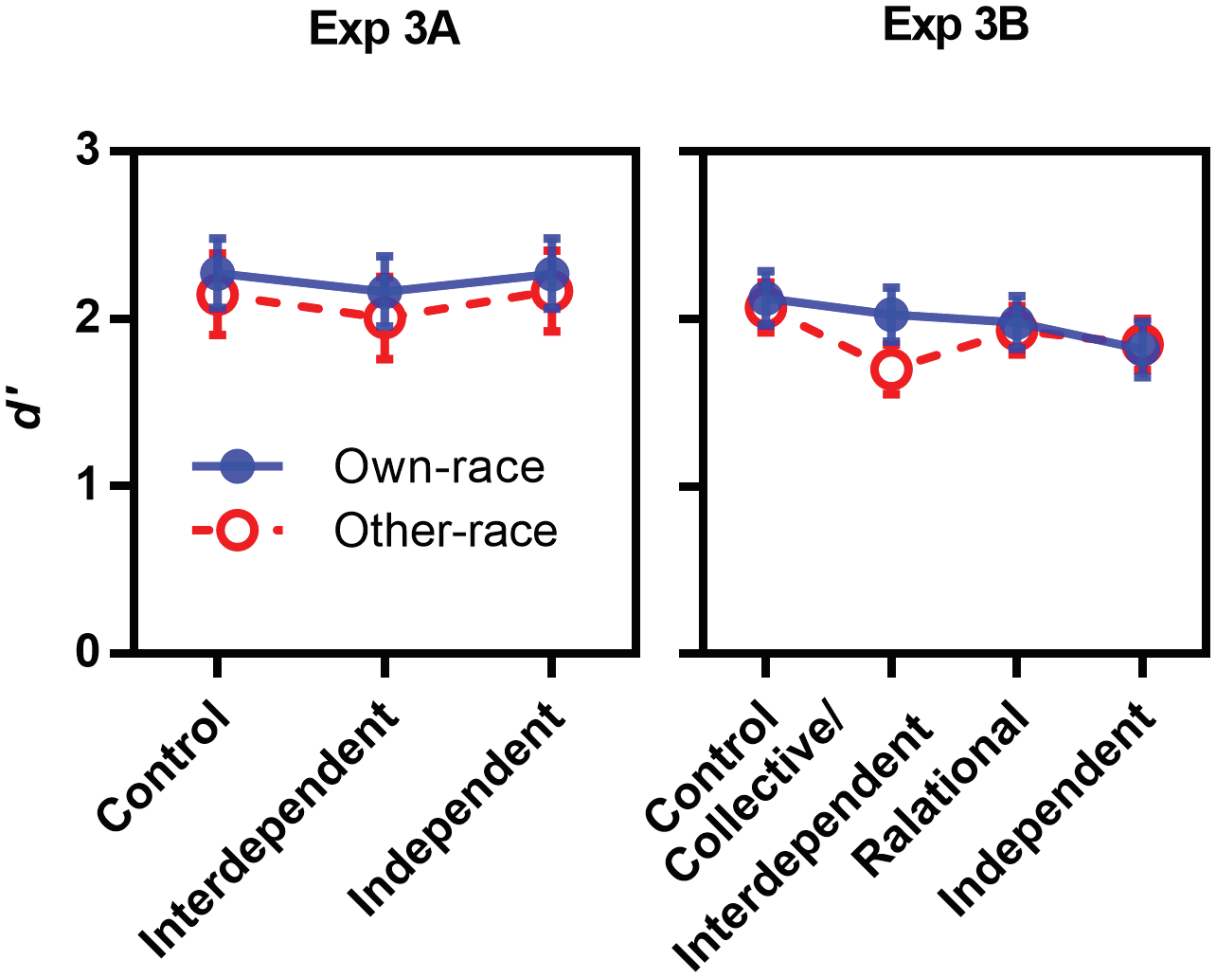
Sensitivity as a function of priming and face race in Experiments 3A and 3B.

Sensitivity *d*′ was submitted to a 4 (priming: control, collective, interdependent, independent) × 2 (face race: own-race, other-race) mixed-design repeated measures ANOVA. Neither main effect nor interaction reached statistical significance (*p*s > 0.26; see Figure 9).

Analysis of hit rates and false alarms also showed no significant effect (*p*s > 0.15).

The results showed no own-race bias, consistent with Fu et al. (2012) and Yi et al. (2015). Normalized face photos may contribute to the null race effect (Yi et al., 2015). We used another set of face stimuli and another priming in Experiment 3A and obtained an own-race bias in FA, also supporting this explanation.

Both Experiments 3A and 3B found no priming effect on own-race/other-race face recognition, consistent with Experiment 2 and Ramon et al. (2016).

##### Eye Movement: Fixation Proportion

**Figure 10:**
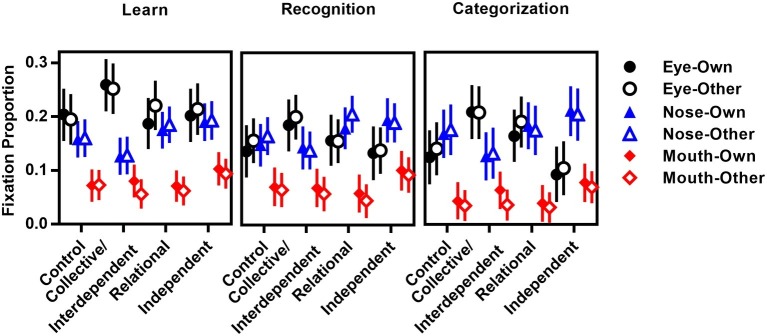
Proportional fixation duration as a function of priming, AOI and race in learning, recognition, and categorization stages in Experiment 3B.

Because participants might scan the face with different total fixation durations under different conditions, in order to examine whether self-construal conditions had a differential influence on fixation patterns on the major facial features, we calculated the proportional fixation duration for each individual AOI area (i.e., eyes, nose, and mouth). Proportional fixation duration was calculated by dividing the total fixation duration within each AOI area by the total fixation duration within the whole face area (see Figure 10 for the proportional fixation duration in each condition).

If the cultural difference of self-construal mirrored the cultural difference of eye movement during face learning, face recognition, and race categorization, an interdependent/collective or relational priming would be expected to increase fixations on nose, while independent priming would increase fixations on eyes.

**Face learning stage**: Proportional fixation duration was submitted to a priming (control, collective/interdependent, relational, independent) × face race (own, other) × AOI (eyes, nose, mouth) mixed-design repeated measure ANOVA. The main effect of AOI was observed, *F*(2,152) = 49.04, *p* < 0.001, $\eta_{p}^{2}$ = 0.39, with the greatest duration of time gazing at eyes (21.7%), then noses (16.4%), and least for mouths (7.6%), *p*s < 0.05. A marginally significant main effect of priming was observed, *F*(3,76) = 2.66, *p = 0*.05, $\eta_{p}^{2}$ = 0.10, with no difference among control, relational, and collective groups, *p*s > 0.41. These three groups tended to spend less amount of time on eyes, nose, and mouth than independent group, *p*s < 0.06. Other main effects or interactions did not reach statistical significance, *p*s > 0.15.

**Face recognition stage**: Similar results were observed in the recognition task. The main effect of AOI was observed, *F*(2,152) = 27.63, *p* < 0.001, $\eta_{p}^{2}$ = 0.27, with roughly equal proportions for eyes (14.8%) and noses (16.0%; *p* = 0.44), and least for mouths (6.6%), *p*s < 0.001. Other main effects or interactions did not reach statistical significance, *p*s > 0.12.

**Race categorization stage**: The main effect of AOI was observed, *F*(2,152) = 29.62, *p* < 0.001, $\eta_{p}^{2}$ = 0.28, with roughly equal proportions for eyes (14.5%) and noses (16.1%; *p* = 0.39), and least for mouths (4.7%), *p*s < 0.001. Importantly, the interaction of AOI with priming was observed, *F*(6,152) = 2.90, *p* = 0.01, $\eta_{p}^{2}$ = 0.10. A simple effect test for eyes showed greater proportional fixation duration for the collective group than the control group (*p* = 0.03) and independent group (*p* = 0.002), and more for the relational group than for the independent group (*p* = 0.025). However, for noses, the fixation duration percentage for the independent group is higher than that of the collective group (*p* = 0.017). Only a tendency was observed for the mouth, with greater fixation duration percentage for the independent group than for the relational group (*p* = 0.091).

During the race categorization stage, AOI interacted with face race, *F*(2,152) = 6.21, *p* = 0.003, $\eta_{p}^{2}$ = 0.08. A simple effect test showed that, both for own-race faces and other-face faces, proportional fixation durations on eyes and noses were longer than for mouths (*p*s < 0.001, no difference between eyes and nose, *p*s > 0.24). However, Chinese participants looked longer at other-race eyes than own-race eyes (*p* = 0.024), less at other-race mouths than own-race mouths (*p* < 0.001), and equally at noses (*p* = 0.95).

Other main effects or interactions did not reach statistical significance, *p*s > 0.34.

In sum, we did not replicate the interaction of AOI and face race during the learning and recognition stages as in Fu et al. (2012) and Yi et al. (2015). This discrepancy may be due to the priming manipulation in our experiment.

We did not observe the priming effect on eye movement during learning or recognition stages, consistent with Ramon et al. (2016). Interestingly, we found this effect during race categorization. We will address detailed discussion in the general discussion section.

## General Discussion

### Self-Construal Priming and Holistic Face Processing

Previous studies have shown that after priming the independent self, the participants are more inclined to analytical processing, and after priming the interdependent self, they tend toward holistic processing (Kühnen and Oyserman, 2002; Lin and Han, 2009; Springer et al., 2012; Liu et al., 2015; Choi et al., 2016). Therefore, we expected to observe increasing holistic processing for interdependent or collective priming and decreasing holistic processing after independent priming.

Our results showed that whether self-construal priming affects holistic face processing depends on the type of holistic processing. That is, the results varied by paradigm. No such priming effect was observed in Experiment 1A with the featural-spacing paradigm. Although the priming effect was observed with the composite paradigm, the patterns were different when using a standard composite paradigm in Experiment 1B and the revised composite paradigm in Experiment 2. Although no priming effect on congruency × alignment holistic processing in Experiment 1B was observed, priming can modulate the congruency effect. As expected, compared with control priming, interdependent priming increased the congruency effect, while independent priming decreased the congruency effect. Experiment 2 also showed a modulation of priming on the congruency effect. However, compared with control priming, no change was observed for any priming, but a larger congruency effect was observed for independent priming than for relational priming, inconsistent with cultural differences in holistic processing.

The inconsistent results of Experiments 1A, 1B, and 2 may come from the different definitions of holistic processing within these different paradigms. It has been shown that composite effects did not correlate with either the part-whole effect (Wang et al., 2012; Rezlescu et al., 2017) or the inversion effect (Rezlescu et al., 2017), which implies that facial holistic processing may reflect distinct facial perceptual mechanisms (Wang et al., 2012; Rezlescu et al., 2017). The standard composite holistic processing used in the present study reflects a tendency to integrate the internal features of a face as a gestalt (Maurer et al., 2002), indexes failure of selective attention to target a facial half while ignoring the other half (Richler et al., 2014), or is only sensitive to objects’ shape information (Zhao et al., 2016a,b). Space-feature holistic processing in our study indexes people’s sensitivity to spatial distances among facial features (Maurer et al., 2002). Therefore, it is very possible that the nature of the standard composite holistic processing used in Experiment 1A, the nature of the space-feature holistic processing used in Experiment 1B, and that of revised composite holistic processing used in Experiment 2 are different and have different sensitivities to self-construal priming.

Similarly, the inconsistent results of the present study and previous object processing research (Kühnen and Oyserman, 2002; Lin and Han, 2009; Springer et al., 2012; Liu et al., 2015; Choi et al., 2016) may also come from the different definitions of holistic processing with these different paradigms. For example, Davidoff et al. (2008) found that Namibians showed significant local processing preferences compared to the British in the Navon perception test. However, in the task of face perception, Namibians have shown a greater face inversion holistic processing effect than the British, indicating that Namibian local processing preferences were not generalized to face-processing tasks. Dale and Arnell (2013) found no relationship among standard Navon task (Navon, 1977) performance, hierarchical shapes in a forced choice task (Kimchi and Palmer, 1982), and superimposed high- and low-pass spatial frequency faces in a forced-choice task (Deruelle et al., 2008). Caparos et al. (2013) proposed that local and global perceptual biases must be distinguished from local and global selective attention. Caparos et al. adopted Navon-like hierarchical figures and asked Namibian subjects who exhibited greater local processing bias than British subjects when making subjective similarity matches regarding hierarchical figures (e.g., Davidoff et al., 2008; Caparos et al., 2012) and British subjects to identify local/global figures while ignoring global/local figures. The authors found that Namibians not only demonstrated a better ability to select local information, but also a better ability to select global information than British subjects.

### Self-Construal Priming and Face Recognition

Present results showed that self-construal priming did not affect own-race face recognition (Experiments 2, 3A, and 3B) or other-race face recognition (Experiments 3A and 3B), consistent with Ramon et al. (2016) and inconsistent with Ng et al. (2015). This discrepancy may be explained by two types of self-construal: chronic self-construal and situational self-construal.

Chronic self-construal refers to a stable personal trait that is mainly influenced by the cultural background and inflexible across varied situations (Markus and Kitayama, 1991). Unlike chronic self-construal, situational self-construal refers to a dominant self-concept according to the immediate situation and is easily activated by priming tasks. A chronically interdependent individual will appear to be independent under certain circumstances and vice versa.

Ng et al. (2015) used the interdependence subscale of the self-construction scale (SCS; Singelis, 1994) to measure the interdependence of European Canadian and East Asian participants. The interdependent orientation that they measured was chronic; whereas, in our study and in Ramon et al. (2016), self-construal was manipulated by priming and was therefore situational. Unlike holistic face processing in Experiments 1A, 1B, and 2 or face orientation processing in Sui and her colleagues’ research (e.g., Sui and Han, 2007; Sui et al., 2013), which could be modulated by situational self-construal, face recognition might be a stable and chronic ability that cannot be situationally affected.

One limitation of the present study is that we did not investigate how the chronic tendency of self-construal of Chinese participants affects their face processing, especially face recognition. Further research with Chinese participants is needed to examine the relationship between chronic self-construal and face processing, especially face recognition, to examine whether the result of Ng et al. (2015) could be replicated.

### Self-Construal Priming and Race Categorization

Based on the cross-cultural studies on eye movement that showed Eastern Asians fixed centrally on the nose region, while Western Caucasians primarily explored the eye and mouth regions during race categorization (e.g., Blais et al., 2008), we expected to find a greater fixation proportion on eyes for the independent priming group and greater nose fixation proportion for the interdependent or collective priming groups. However, we found a self-construal priming effect on eye movements during race categorization. Specifically, for eyes, a greater fixation proportion was noted for the collective group than for the control group, and greater for the interdependent group than for the independent group. However, for the nose, a greater fixation proportion was observed for the independent than for the collective group. Only a tendency was observed for mouths, with greater fixation proportion for the independent group than for the interdependent group. These results are inconsistent with our prediction.

The results of relational self-construal and collective/interdependent self-construal have some similarities, which are different from the results of independent self-construal. The relational self and the collective/interdependent self are more inclined to engage with others. Holland et al. (2004) found that self-construal activation automatically influences interpersonal behavior as reflected in the actual distance between the self and others. Therefore, it is no surprise that relational priming or collective priming showed more eye fixation and less nose fixation than independent priming, given that these people were more inclined to interact with others and more inclined to eye contact; after independent priming, people are less likely to make eye contact with others.

### Conclusion and Limitation

In summary, our study adopted several face recognition paradigms, several holistic face processing paradigms, and several self-construal priming tasks to investigate the effects of self-construal priming on face recognition, holistic face processing, and race categorization. The results showed that self-construal priming had no effect on face recognition, but had varied influence on holistic face processing depending on the holistic processing paradigm and had an effect on race categorization in eye movement. These results indicated that cultural differences in self-construal could not simply mirror cultural differences in face processing.

We are confident of these results since several experiments in the present study have obtained consistent results, which were also consistent with previous studies (e.g., Ramon et al., 2016). However, the present study could not rule out the possibility that the null results in the present study were due to not enough power to test the not strong enough priming effect on face processing. For example, from Figures 2, 9, and 10, we can observe some weak although not significant effect of self-construal priming on face processing. Present study only took an initial step to investigate the effect of self-construal priming on holistic face processing, face recognition, and race categorization. Future studies are encouraged to adopt more participants, more powerful priming task (e.g., story-reading task), and more sensitive measurement (e.g., using eye movement to measure priming effect on holistic face processing) to examine and extend present findings.

## Data Availability

All datasets generated for this study are included in the manuscript and/or the supplementary files.

## Ethics Statement

This study was carried out in accordance with the recommendations of “protection of human participants, Institutional Review Board of Department of Psychology at Sun Yat-sen University” with written informed consent from all subjects. All subjects gave written informed consent in accordance with the Declaration of Helsinki. The protocol was approved by the Institutional Review Board of Department of Psychology at Sun Yat-sen University.

## Author Contributions

GZ developed the concept. GZ and XLi developed the design of the whole experimental work. CF and XLia developed the design of the Experiment 3B. XLi, XLia, CF, and GZ actively participated in the implementation of the experimental tasks, data collection, and data analyses in the experiments. All authors contributed to writing and reviewing the manuscript and approved the final version of the manuscript.

### Conflict of Interest Statement

The authors declare that the research was conducted in the absence of any commercial or financial relationships that could be construed as a potential conflict of interest.
